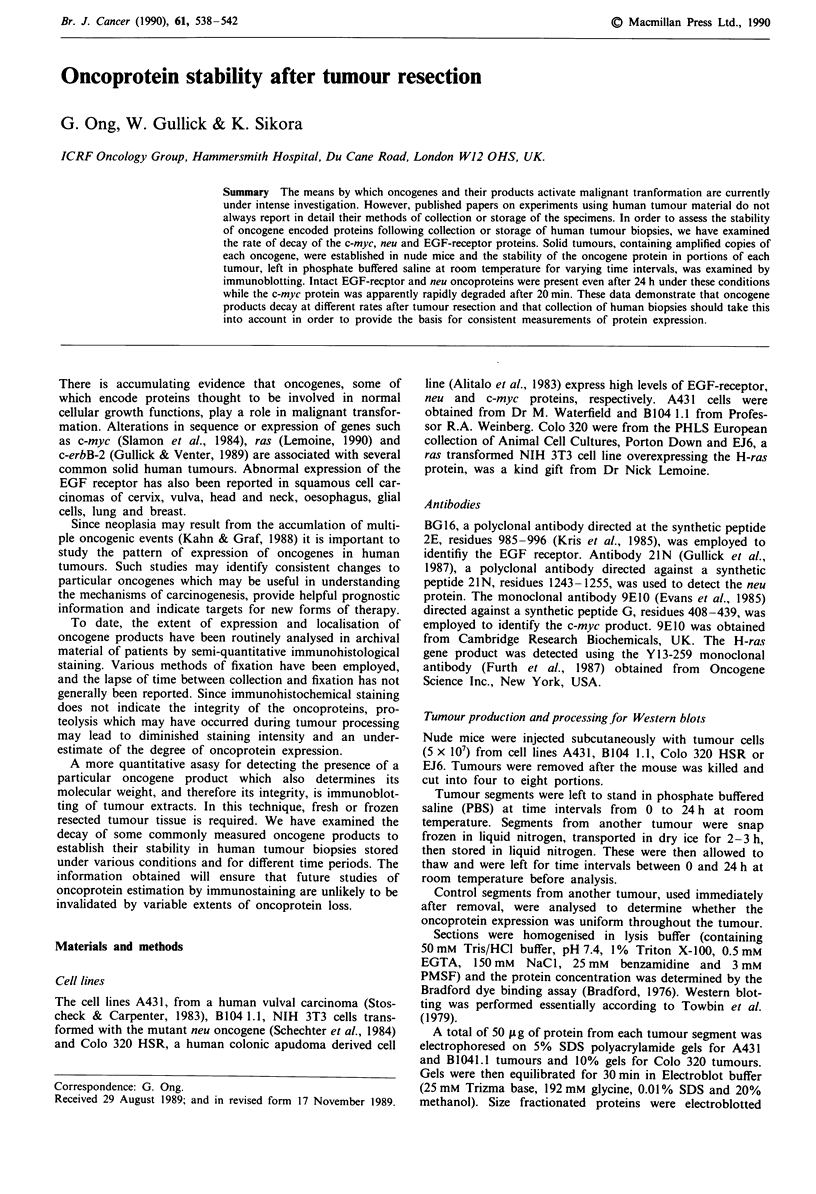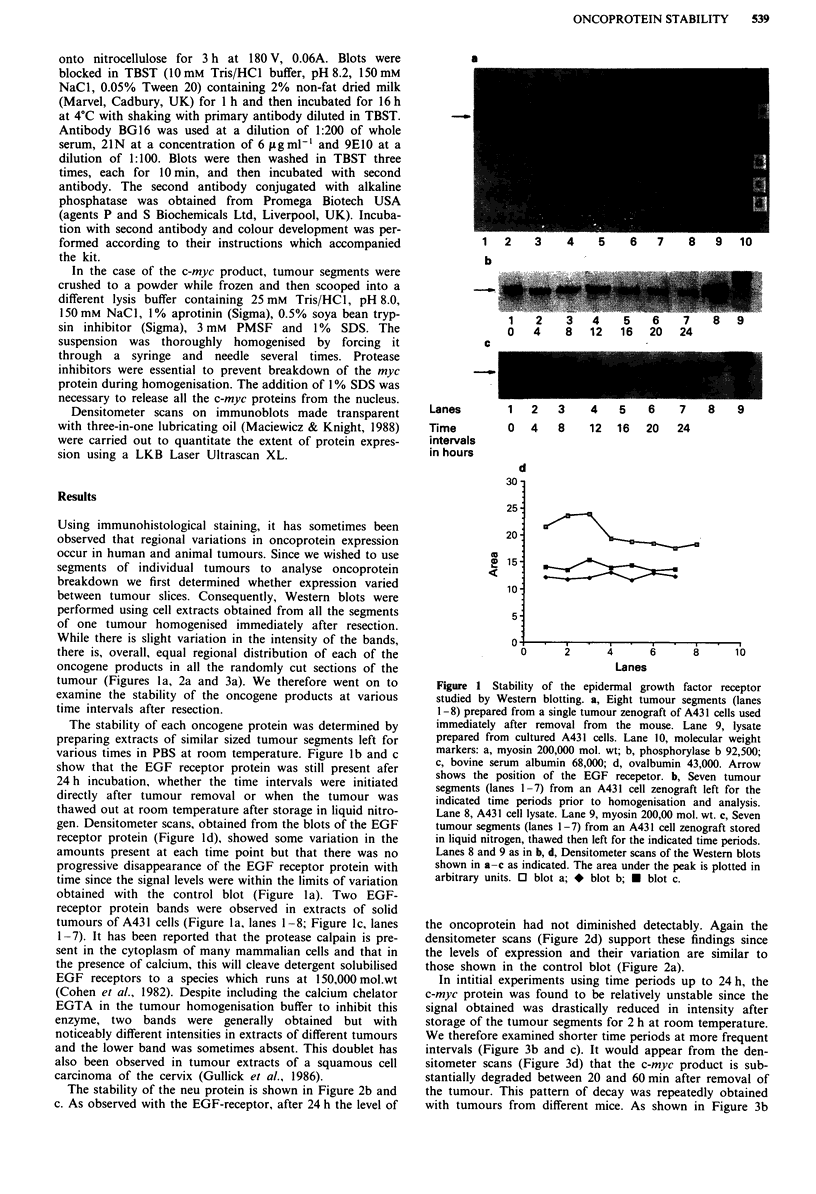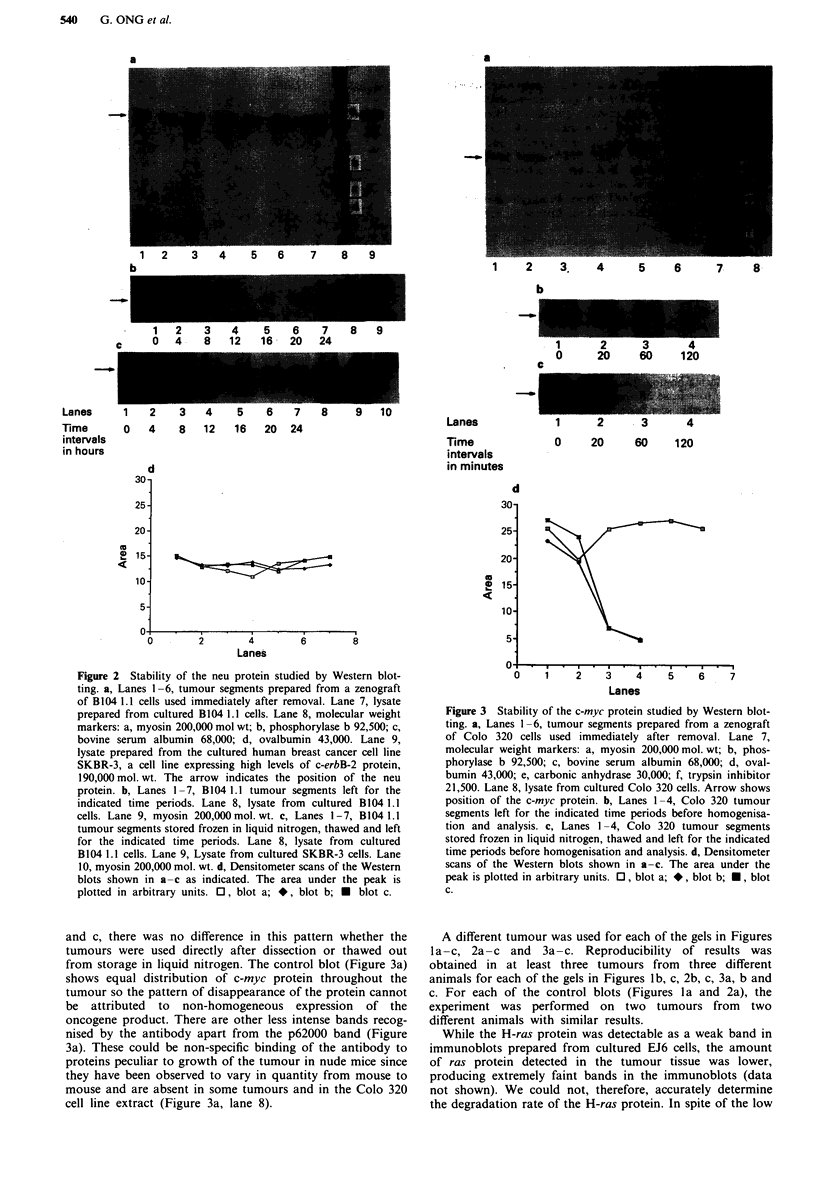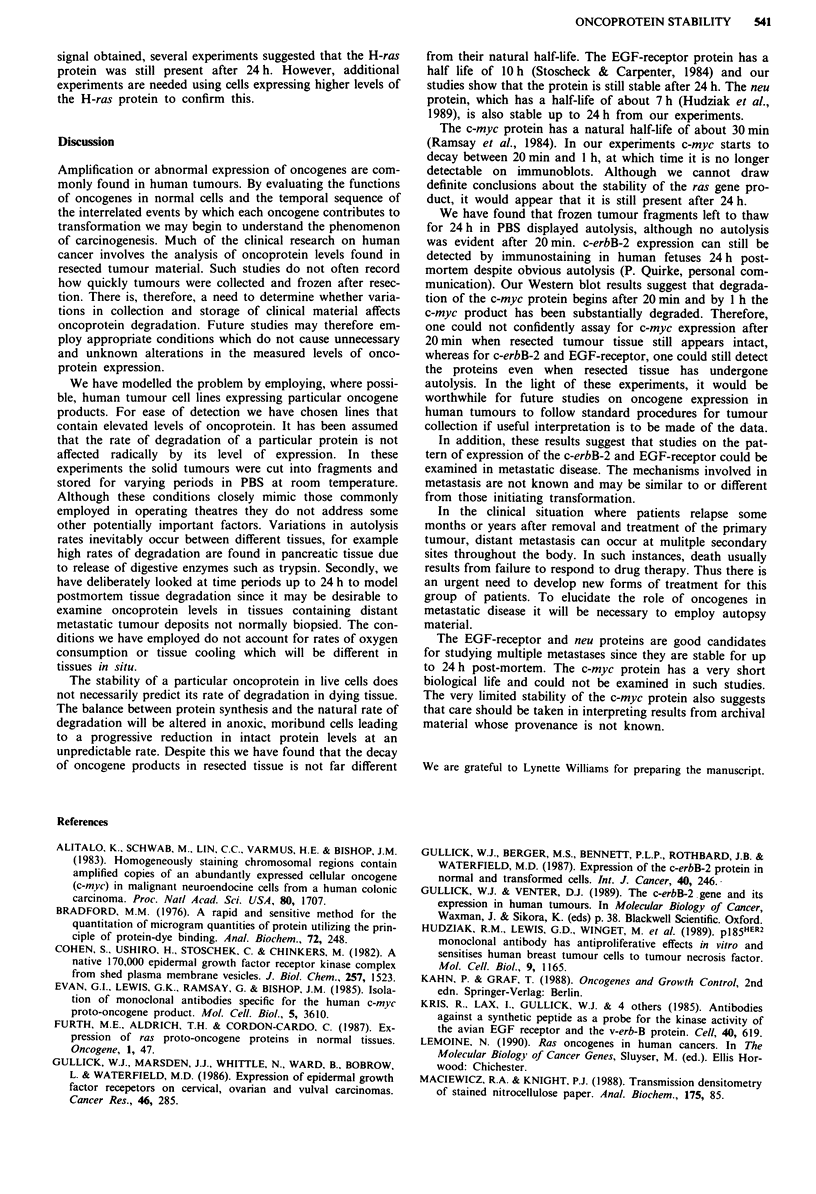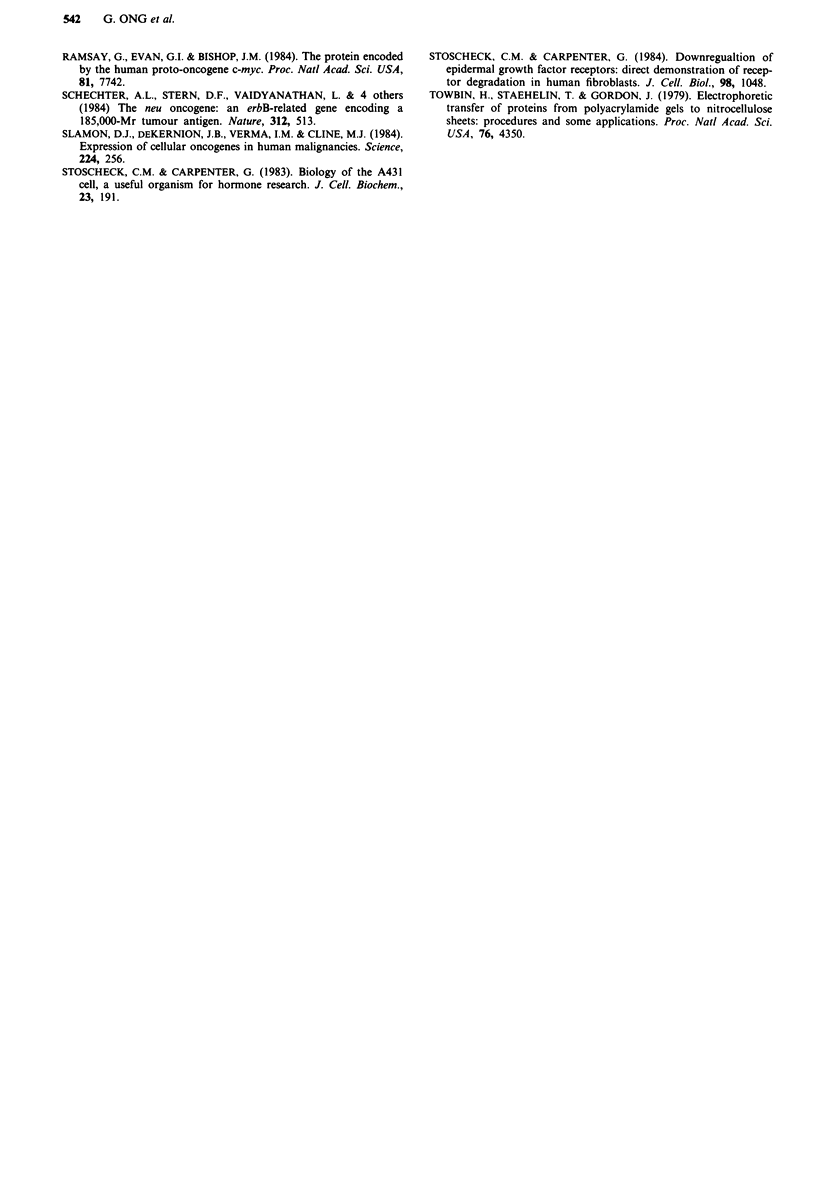# Oncoprotein stability after tumour resection.

**DOI:** 10.1038/bjc.1990.121

**Published:** 1990-04

**Authors:** G. Ong, W. Gullick, K. Sikora

**Affiliations:** ICRF Oncology Group, Hammersmith Hospital, London, UK.

## Abstract

**Images:**


Br. J. Cancer (1990), 61, 538-542 © Macmillan Press Ltd., 1990

### Oncoprotein stability after tumour resection

G. Ong, W. Gullick & K. Sikora

*ICRF Oncology Group, Hammersmith Hospital, Du Cane Road, London W12 OHS, UK.*

**Summary** The means by which oncogenes and their products activate malignant tranformation are currently under intense investigation. However, published papers on experiments using human tumour material do not always report in detail their methods of collection or storage of the specimens. In order to assess the stability of oncogene encoded proteins following collection or storage of human tumour biopsies, we have examined the rate of decay of the c-*myc*, *neu* and EGF-receptor proteins. Solid tumours, containing amplified copies of each oncogene, were established in nude mice and the stability of the oncogene protein in portions of each tumour, left in phosphate buffered saline at room temperature for varying time intervals, was examined by immunoblotting. Intact EGF-recptor and *neu* oncoproteins were present even after 24 h under these conditions while the c-*myc* protein was apparently rapidly degraded after 20 min. These data demonstrate that oncogene products decay at different rates after tumour resection and that collection of human biopsies should take this into account in order to provide the basis for consistent measurements of protein expression.

There is accumulating evidence that oncogenes, some of which encode proteins thought to be involved in normal cellular growth functions, play a role in malignant transformation. Alterations in sequence or expression of genes such as c-*myc* (Slamon *et al.*, 1984), *ras* (Lemoine, 1990) and c-*erb*B-2 (Gullick & Venter, 1989) are associated with several common solid human tumours. Abnormal expression of the EGF receptor has also been reported in squamous cell carcinomas of cervix, vulva, head and neck, oesophagus, glial cells, lung and breast.

Since neoplasia may result from the accumlation of multiple oncogenic events (Kahn & Graf, 1988) it is important to study the pattern of expression of oncogenes in human tumours. Such studies may identify consistent changes to particular oncogenes which may be useful in understanding the mechanisms of carcinogenesis, provide helpful prognostic information and indicate targets for new forms of therapy.

To date, the extent of expression and localisation of oncogene products have been routinely analysed in archival material of patients by semi-quantitative immunohistological staining. Various methods of fixation have been employed, and the lapse of time between collection and fixation has not generally been reported. Since immunohistochemical staining does not indicate the integrity of the oncoproteins, proteolysis which may have occurred during tumour processing may lead to diminished staining intensity and an underestimate of the degree of oncoprotein expression.

A more quantitative asasy for detecting the presence of a particular oncogene product which also determines its molecular weight, and therefore its integrity, is immunoblotting of tumour extracts. In this technique, fresh or frozen resected tumour tissue is required. We have examined the decay of some commonly measured oncogene products to establish their stability in human tumour biopsies stored under various conditions and for different time periods. The information obtained will ensure that future studies of oncoprotein estimation by immunostaining are unlikely to be invalidated by variable extents of oncoprotein loss.

### Materials and methods

### *Cell lines*

The cell lines A431, from a human vulval carcinoma (Stoscheck & Carpenter, 1983), B104 1.1, NIH 3T3 cells transformed with the mutant *neu* oncogene (Schechter *et al.*, 1984) and Colo 320 HSR, a human colonic apudoma derived cell line (Alitalo *et al.*, 1983) express high levels of EGF-receptor, *neu* and c-*myc* proteins, respectively. A431 cells were obtained from Dr M. Waterfield and B104 1.1 from Professor R.A. Weinberg. Colo 320 were from the PHLS European collection of Animal Cell Cultures, Porton Down and EJ6, a *ras* transformed NIH 3T3 cell line overexpressing the H-*ras* protein, was a kind gift from Dr Nick Lemoine.

### *Antibodies*

BG16, a polyclonal antibody directed at the synthetic peptide 2E, residues 985-996 (Kris *et al.*, 1985), was employed to identifiy the EGF receptor. Antibody 21N (Gullick *et al.*, 1987), a polyclonal antibody directed against a synthetic peptide 21N, residues 1243-1255, was used to detect the *neu* protein. The monoclonal antibody 9E10 (Evans *et al.*, 1985) directed against a synthetic peptide G, residues 408-439, was employed to identify the c-*myc* product. 9E10 was obtained from Cambridge Research Biochemicals, UK. The H-*ras* gene product was detected using the Y13-259 monoclonal antibody (Furth *et al.*, 1987) obtained from Oncogene Science Inc., New York, USA.

### *Tumour production and processing for Western blots*

Nude mice were injected subcutaneously with tumour cells ($5 \times 10^7$) from cell lines A431, B104 1.1, Colo 320 HSR or EJ6. Tumours were removed after the mouse was killed and cut into four to eight portions.

Tumour segments were left to stand in phosphate buffered saline (PBS) at time intervals from 0 to 24 h at room temperature. Segments from another tumour were snap frozen in liquid nitrogen, transported in dry ice for 2-3 h, then stored in liquid nitrogen. These were then allowed to thaw and were left for time intervals between 0 and 24 h at room temperature before analysis.

Control segments from another tumour, used immediately after removal, were analysed to determine whether the oncoprotein expression was uniform throughout the tumour.

Sections were homogenised in lysis buffer (containing 50 mM Tris/HCl buffer, pH 7.4, 1% Triton X-100, 0.5 mM EGTA, 150 mM NaCl, 25 mM benzamidine and 3 mM PMSF) and the protein concentration was determined by the Bradford dye binding assay (Bradford, 1976). Western blotting was performed essentially according to Towbin *et al.* (1979).

A total of 50 µg of protein from each tumour segment was electrophoresed on 5% SDS polyacrylamide gels for A431 and B1041.1 tumours and 10% gels for Colo 320 tumours. Gels were then equilibrated for 30 min in Electroblot buffer (25 mM Trizma base, 192 mM glycine, 0.01% SDS and 20% methanol). Size fractionated proteins were electroblotted

Correspondence: G. Ong.
Received 29 August 1989; and in revised form 17 November 1989.

onto nitrocellulose for 3 h at 180 V, 0.06A. Blots were blocked in TBST (10 mM Tris/HCl buffer, pH 8.2, 150 mM NaCl, 0.05% Tween 20) containing 2% non-fat dried milk (Marvel, Cadbury, UK) for 1 h and then incubated for 16 h at 4°C with shaking with primary antibody diluted in TBST. Antibody BG16 was used at a dilution of 1:200 of whole serum, 21N at a concentration of 6 $\mu g\ ml^{-1}$ and 9E10 at a dilution of 1:100. Blots were then washed in TBST three times, each for 10 min, and then incubated with second antibody. The second antibody conjugated with alkaline phosphatase was obtained from Promega Biotech USA (agents P and S Biochemicals Ltd, Liverpool, UK). Incubation with second antibody and colour development was performed according to their instructions which accompanied the kit.

In the case of the c-*myc* product, tumour segments were crushed to a powder while frozen and then scooped into a different lysis buffer containing 25 mM Tris/HCl, pH 8.0, 150 mM NaCl, 1% aprotinin (Sigma), 0.5% soya bean trypsin inhibitor (Sigma), 3 mM PMSF and 1% SDS. The suspension was thoroughly homogenised by forcing it through a syringe and needle several times. Protease inhibitors were essential to prevent breakdown of the *myc* protein during homogenisation. The addition of 1% SDS was necessary to release all the c-*myc* proteins from the nucleus.

Densitometer scans on immunoblots made transparent with three-in-one lubricating oil (Maciewicz & Knight, 1988) were carried out to quantitate the extent of protein expression using a LKB Laser Ultrascan XL.

### Results

Using immunohistological staining, it has sometimes been observed that regional variations in oncoprotein expression occur in human and animal tumours. Since we wished to use segments of individual tumours to analyse oncoprotein breakdown we first determined whether expression varied between tumour slices. Consequently, Western blots were performed using cell extracts obtained from all the segments of one tumour homogenised immediately after resection. While there is slight variation in the intensity of the bands, there is, overall, equal regional distribution of each of the oncogene products in all the randomly cut sections of the tumour (Figures 1a, 2a and 3a). We therefore went on to examine the stability of the oncogene products at various time intervals after resection.

The stability of each oncogene protein was determined by preparing extracts of similar sized tumour segments left for various times in PBS at room temperature. Figure 1b and c show that the EGF receptor protein was still present afer 24 h incubation, whether the time intervals were initiated directly after tumour removal or when the tumour was thawed out at room temperature after storage in liquid nitrogen. Densitometer scans, obtained from the blots of the EGF receptor protein (Figure 1d), showed some variation in the amounts present at each time point but that there was no progressive disappearance of the EGF receptor protein with time since the signal levels were within the limits of variation obtained with the control blot (Figure 1a). Two EGF-receptor protein bands were observed in extracts of solid tumours of A431 cells (Figure 1a, lanes 1–8; Figure 1c, lanes 1–7). It has been reported that the protease calpain is present in the cytoplasm of many mammalian cells and that in the presence of calcium, this will cleave detergent solubilised EGF receptors to a species which runs at 150,000 mol.wt (Cohen *et al*., 1982). Despite including the calcium chelator EGTA in the tumour homogenisation buffer to inhibit this enzyme, two bands were generally obtained but with noticeably different intensities in extracts of different tumours and the lower band was sometimes absent. This doublet has also been observed in tumour extracts of a squamous cell carcinoma of the cervix (Gullick *et al*., 1986).

The stability of the neu protein is shown in Figure 2b and c. As observed with the EGF-receptor, after 24 h the level of

**Figure 1** Stability of the epidermal growth factor receptor studied by Western blotting. **a**, Eight tumour segments (lanes 1–8) prepared from a single tumour zenograft of A431 cells used immediately after removal from the mouse. Lane 9, lysate prepared from cultured A431 cells. Lane 10, molecular weight markers: a, myosin 200,000 mol. wt; b, phosphorylase b 92,500; c, bovine serum albumin 68,000; d, ovalbumin 43,000. Arrow shows the position of the EGF recepetor. **b**, Seven tumour segments (lanes 1–7) from an A431 cell zenograft left for the indicated time periods prior to homogenisation and analysis. Lane 8, A431 cell lysate. Lane 9, myosin 200,00 mol. wt. **c**, Seven tumour segments (lanes 1–7) from an A431 cell zenograft stored in liquid nitrogen, thawed then left for the indicated time periods. Lanes 8 and 9 as in **b**, **d**, Densitometer scans of the Western blots shown in **a**–**c** as indicated. The area under the peak is plotted in arbitrary units. □ blot a; ◆ blot b; ■ blot c.

the oncoprotein had not diminished detectably. Again the densitometer scans (Figure 2d) support these findings since the levels of expression and their variation are similar to those shown in the control blot (Figure 2a).

In intitial experiments using time periods up to 24 h, the c-*myc* protein was found to be relatively unstable since the signal obtained was drastically reduced in intensity after storage of the tumour segments for 2 h at room temperature. We therefore examined shorter time periods at more frequent intervals (Figure 3b and c). It would appear from the densitometer scans (Figure 3d) that the c-*myc* product is substantially degraded between 20 and 60 min after removal of the tumour. This pattern of decay was repeatedly obtained with tumours from different mice. As shown in Figure 3b

**Figure 2** Stability of the neu protein studied by Western blotting. **a**, Lanes 1–6, tumour segments prepared from a zenograft of B104 1.1 cells used immediately after removal. Lane 7, lysate prepared from cultured B104 1.1 cells. Lane 8, molecular weight markers: a, myosin 200,000 mol wt; b, phosphorylase b 92,500; c, bovine serum albumin 68,000; d, ovalbumin 43,000. Lane 9, lysate prepared from the cultured human breast cancer cell line SKBR-3, a cell line expressing high levels of c-*erb*B-2 protein, 190,000 mol. wt. The arrow indicates the position of the neu protein. **b**, Lanes 1–7, B104 1.1 tumour segments left for the indicated time periods. Lane 8, lysate from cultured B104 1.1 cells. Lane 9, myosin 200,000 mol. wt. **c**, Lanes 1–7, B104 1.1 tumour segments stored frozen in liquid nitrogen, thawed and left for the indicated time periods. Lane 8, lysate from cultured B104 1.1 cells. Lane 9, Lysate from cultured SKBR-3 cells. Lane 10, myosin 200,000 mol. wt. **d**, Densitometer scans of the Western blots shown in **a**–**c** as indicated. The area under the peak is plotted in arbitrary units. □, blot a; ◆, blot b; ■ blot c.

**Figure 3** Stability of the c-*myc* protein studied by Western blotting. **a**, Lanes 1–6, tumour segments prepared from a zenograft of Colo 320 cells used immediately after removal. Lane 7, molecular weight markers: a, myosin 200,000 mol. wt; b, phosphorylase b 92,500; c, bovine serum albumin 68,000; d, ovalbumin 43,000; e, carbonic anhydrase 30,000; f, trypsin inhibitor 21,500. Lane 8, lysate from cultured Colo 320 cells. Arrow shows position of the c-*myc* protein. **b**, Lanes 1–4, Colo 320 tumour segments left for the indicated time periods before homogenisation and analysis. **c**, Lanes 1–4, Colo 320 tumour segments stored frozen in liquid nitrogen, thawed and left for the indicated time periods before homogenisation and analysis. **d**, Densitometer scans of the Western blots shown in **a**–**c**. The area under the peak is plotted in arbitrary units. □, blot a; ◆, blot b; ■, blot c.

and c, there was no difference in this pattern whether the tumours were used directly after dissection or thawed out from storage in liquid nitrogen. The control blot (Figure 3a) shows equal distribution of c-*myc* protein throughout the tumour so the pattern of disappearance of the protein cannot be attributed to non-homogeneous expression of the oncogene product. There are other less intense bands recognised by the antibody apart from the p62000 band (Figure 3a). These could be non-specific binding of the antibody to proteins peculiar to growth of the tumour in nude mice since they have been observed to vary in quantity from mouse to mouse and are absent in some tumours and in the Colo 320 cell line extract (Figure 3a, lane 8).

A different tumour was used for each of the gels in Figures 1a–c, 2a–c and 3a–c. Reproducibility of results was obtained in at least three tumours from three different animals for each of the gels in Figures 1b, c, 2b, c, 3a, b and c. For each of the control blots (Figures 1a and 2a), the experiment was performed on two tumours from two different animals with similar results.

While the H-*ras* protein was detectable as a weak band in immunoblots prepared from cultured EJ6 cells, the amount of *ras* protein detected in the tumour tissue was lower, producing extremely faint bands in the immunoblots (data not shown). We could not, therefore, accurately determine the degradation rate of the H-*ras* protein. In spite of the low signal obtained, several experiments suggested that the H-*ras* protein was still present after 24 h. However, additional experiments are needed using cells expressing higher levels of the H-*ras* protein to confirm this.

### Discussion

Amplification or abnormal expression of oncogenes are commonly found in human tumours. By evaluating the functions of oncogenes in normal cells and the temporal sequence of the interrelated events by which each oncogene contributes to transformation we may begin to understand the phenomenon of carcinogenesis. Much of the clinical research on human cancer involves the analysis of oncoprotein levels found in resected tumour material. Such studies do not often record how quickly tumours were collected and frozen after resection. There is, therefore, a need to determine whether variations in collection and storage of clinical material affects oncoprotein degradation. Future studies may therefore employ appropriate conditions which do not cause unnecessary and unknown alterations in the measured levels of oncoprotein expression.

We have modelled the problem by employing, where possible, human tumour cell lines expressing particular oncogene products. For ease of detection we have chosen lines that contain elevated levels of oncoprotein. It has been assumed that the rate of degradation of a particular protein is not affected radically by its level of expression. In these experiments the solid tumours were cut into fragments and stored for varying periods in PBS at room temperature. Although these conditions closely mimic those commonly employed in operating theatres they do not address some other potentially important factors. Variations in autolysis rates inevitably occur between different tissues, for example high rates of degradation are found in pancreatic tissue due to release of digestive enzymes such as trypsin. Secondly, we have deliberately looked at time periods up to 24 h to model postmortem tissue degradation since it may be desirable to examine oncoprotein levels in tissues containing distant metastatic tumour deposits not normally biopsied. The conditions we have employed do not account for rates of oxygen consumption or tissue cooling which will be different in tissues *in situ*.

The stability of a particular oncoprotein in live cells does not necessarily predict its rate of degradation in dying tissue. The balance between protein synthesis and the natural rate of degradation will be altered in anoxic, moribund cells leading to a progressive reduction in intact protein levels at an unpredictable rate. Despite this we have found that the decay of oncogene products in resected tissue is not far different from their natural half-life. The EGF-receptor protein has a half life of 10 h (Stoscheck & Carpenter, 1984) and our studies show that the protein is still stable after 24 h. The *neu* protein, which has a half-life of about 7 h (Hudziak *et al.*, 1989), is also stable up to 24 h from our experiments.

The c-*myc* protein has a natural half-life of about 30 min (Ramsay *et al.*, 1984). In our experiments c-*myc* starts to decay between 20 min and 1 h, at which time it is no longer detectable on immunoblots. Although we cannot draw definite conclusions about the stability of the *ras* gene product, it would appear that it is still present after 24 h.

We have found that frozen tumour fragments left to thaw for 24 h in PBS displayed autolysis, although no autolysis was evident after 20 min. c-*erb*B-2 expression can still be detected by immunostaining in human fetuses 24 h post-mortem despite obvious autolysis (P. Quirke, personal communication). Our Western blot results suggest that degradation of the c-*myc* protein begins after 20 min and by 1 h the c-*myc* product has been substantially degraded. Therefore, one could not confidently assay for c-*myc* expression after 20 min when resected tumour tissue still appears intact, whereas for c-*erb*B-2 and EGF-receptor, one could still detect the proteins even when resected tissue has undergone autolysis. In the light of these experiments, it would be worthwhile for future studies on oncogene expression in human tumours to follow standard procedures for tumour collection if useful interpretation is to be made of the data.

In addition, these results suggest that studies on the pattern of expression of the c-*erb*B-2 and EGF-receptor could be examined in metastatic disease. The mechanisms involved in metastasis are not known and may be similar to or different from those initiating transformation.

In the clinical situation where patients relapse some months or years after removal and treatment of the primary tumour, distant metastasis can occur at mulitple secondary sites throughout the body. In such instances, death usually results from failure to respond to drug therapy. Thus there is an urgent need to develop new forms of treatment for this group of patients. To elucidate the role of oncogenes in metastatic disease it will be necessary to employ autopsy material.

The EGF-receptor and *neu* proteins are good candidates for studying multiple metastases since they are stable for up to 24 h post-mortem. The c-*myc* protein has a very short biological life and could not be examined in such studies. The very limited stability of the c-*myc* protein also suggests that care should be taken in interpreting results from archival material whose provenance is not known.

We are grateful to Lynette Williams for preparing the manuscript.

### References

ALITALO, K., SCHWAB, M., LIN, C.C., VARMUS, H.E. & BISHOP, J.M. (1983). Homogeneously staining chromosomal regions contain amplified copies of an abundantly expressed cellular oncogene (c-*myc*) in malignant neuroendocine cells from a human colonic carcinoma. *Proc. Natl Acad. Sci. USA*, **80**, 1707.

BRADFORD, M.M. (1976). A rapid and sensitive method for the quantitation of microgram quantities of protein utilizing the principle of protein-dye binding. *Anal. Biochem.*, **72**, 248.

COHEN, S., USHIRO, H., STOSCHEK, C. & CHINKERS, M. (1982). A native 170,000 epidermal growth factor receptor kinase complex from shed plasma membrane vesicles. *J. Biol. Chem.*, **257**, 1523.

EVAN, G.I., LEWIS, G.K., RAMSAY, G. & BISHOP, J.M. (1985). Isolation of monoclonal antibodies specific for the human c-*myc* proto-oncogene product. *Mol. Cell. Biol.*, **5**, 3610.

FURTH, M.E., ALDRICH, T.H. & CORDON-CARDO, C. (1987). Expression of *ras* proto-oncogene proteins in normal tissues. *Oncogene*, **1**, 47.

GULLICK, W.J., MARSDEN, J.J., WHITTLE, N., WARD, B., BOBROW, L. & WATERFIELD, M.D. (1986). Expression of epidermal growth factor recepetors on cervical, ovarian and vulval carcinomas. *Cancer Res.*, **46**, 285.

GULLICK, W.J., BERGER, M.S., BENNETT, P.L.P., ROTHBARD, J.B. & WATERFIELD, M.D. (1987). Expression of the c-*erb*B-2 protein in normal and transformed cells. *Int. J. Cancer*, **40**, 246.

GULLICK, W.J. & VENTER, D.J. (1989). The c-*erb*B-2 gene and its expression in human tumours. In *Molecular Biology of Cancer*, Waxman, J. & Sikora, K. (eds) p. 38. Blackwell Scientific. Oxford.

HUDZIAK, R.M., LEWIS, G.D., WINGET, M. *et al.* (1989). $p185^{HER2}$ monoclonal antibody has antiproliferative effects *in vitro* and sensitises human breast tumour cells to tumour necrosis factor. *Mol. Cell. Biol.*, **9**, 1165.

KAHN, P. & GRAF, T. (1988). *Oncogenes and Growth Control*, 2nd edn. Springer-Verlag: Berlin.

KRIS, R., LAX, I., GULLICK, W.J. & 4 others (1985). Antibodies against a synthetic peptide as a probe for the kinase activity of the avian EGF receptor and the v-*erb*-B protein. *Cell*, **40**, 619.

LEMOINE, N. (1990). *Ras* oncogenes in human cancers. In *The Molecular Biology of Cancer Genes*, Sluyser, M. (ed.). Ellis Horwood: Chichester.

MACIEWICZ, R.A. & KNIGHT, P.J. (1988). Transmission densitometry of stained nitrocellulose paper. *Anal. Biochem.*, **175**, 85.

RAMSAY, G., EVAN, G.I. & BISHOP, J.M. (1984). The protein encoded by the human proto-oncogene c-*myc*. *Proc. Natl Acad. Sci. USA*, **81**, 7742.

SCHECHTER, A.L., STERN, D.F., VAIDYANATHAN, L. & 4 others (1984) The *neu* oncogene: an *erb*B-related gene encoding a 185,000-Mr tumour antigen. *Nature*, **312**, 513.

SLAMON, D.J., DEKERNION, J.B., VERMA, I.M. & CLINE, M.J. (1984). Expression of cellular oncogenes in human malignancies. *Science*, **224**, 256.

STOSCHECK, C.M. & CARPENTER, G. (1983). Biology of the A431 cell, a useful organism for hormone research. *J. Cell. Biochem.*, **23**, 191.

STOSCHECK, C.M. & CARPENTER, G. (1984). Downregualtion of epidermal growth factor receptors: direct demonstration of receptor degradation in human fibroblasts. *J. Cell. Biol.*, **98**, 1048.

TOWBIN, H., STAEHELIN, T. & GORDON, J. (1979). Electrophoretic transfer of proteins from polyacrylamide gels to nitrocellulose sheets: procedures and some applications. *Proc. Natl Acad. Sci. USA*, **76**, 4350.